# Removal of Zinc from Aqueous Solution by Optimized Oil Palm Empty Fruit Bunches Biochar as Low Cost Adsorbent

**DOI:** 10.1155/2017/7914714

**Published:** 2017-03-21

**Authors:** Seyed Ali Zamani, Robiah Yunus, A. W. Samsuri, M. A. Mohd Salleh, Bahareh Asady

**Affiliations:** ^1^Department of Civil Engineering, Faculty of Engineering, Universiti Putra Malaysia, 43300 UPM Serdang, Selangor, Malaysia; ^2^Department of Chemical and Environmental Engineering, Faculty of Engineering, Universiti Putra Malaysia, 43300 UPM Serdang, Selangor, Malaysia; ^3^Department of Land Management, Faculty of Agriculture, Universiti Putra Malaysia, 43400 UPM Serdang, Selangor, Malaysia; ^4^Institute of Advanced Technology, Faculty of Engineering, Universiti Putra Malaysia, 43400 UPM Serdang, Selangor, Malaysia

## Abstract

This study aims to produce optimized biochar from oil palm empty fruit bunches (OPEFB), as a green, low cost adsorbent for uptake of zinc from aqueous solution. The impact of pyrolysis conditions, namely, highest treatment temperature (HTT), heating rate (HR), and residence time (RT) on biochar yield and adsorption capacity towards zinc, was investigated. Mathematical modeling and optimization of independent variables were performed employing response surface methodology (RSM). HTT was found to be the most influential variable, followed by residence time and heating rate. Based on the central composite design (CCD), two quadratic models were developed to correlate three independent variables to responses. The optimum production condition for OPEFB biochar was found as follows: HTT of 615°C, HR of 8°C/min, and RT of 128 minutes. The optimum biochar showed 15.18 mg/g adsorption capacity for zinc and 25.49% of yield which was in agreement with the predicted values, satisfactory. Results of the characterization of optimum product illustrated well-developed BET surface area and porous structure in optimum product which favored its sorptive ability.

## 1. Introduction

Heavy metal pollution is one of the most serious environmental issues, faced worldwide today [1–3]. Intense industrial activities result in releasing of significant amount of heavy metals including zinc into aqua environment [4]. Mining, metal coating, galvanizing steel and iron, and production of batteries, as well as production of deodorants, paints, ceramic, wood, drug, and fabrics, are major industries, responsible for releasing of zinc into environment [4]. Human exposure to intense amount of zinc leads to metal-fume fever, vomiting, stomach cramp, nausea, loss of appetite, and neurological signs such as ataxia [5]. Health risks of zinc contaminated water for both human and other living organisms is a great concern because of its non biodegradability and mobility. According to World Health Organization guidelines for quality of drinking water and to protect the environment, maximum acceptable zinc level in drinking water is recommended as 5.00 mg/l [6]. Due to global shortage of water resources, health risks, and environmental problems associated with heavy metal pollution such as zinc, treatment of wastewater by effective methods is critical.

Different methods and techniques have been introduced for controlling water and wastewater pollution by heavy metals such as ion exchange, adsorption, precipitation, coagulation, membrane technologies, and reverse osmosis. Among these methodologies, adsorption has been demonstrated to be an economically practicable alternative method for uptake of metals from water system [7, 8]. The simplicity of operation and design in this method as well as its effectiveness in minimizing various types of pollutants leads to its widespread applicability in controlling water pollution [9]. High cost of adsorption by commercially produced activated carbon leads to extensive researches on possibility of using waste biomaterials, as low cost sorbents, for treatment of water and wastewater from heavy metal contaminants [10].

In recent years, biochar from different agrobased and municipal waste materials has been shown to be a potential low cost alternative for separation of heavy metal from water system [11, 12]. Different agrobased and municipal wastes derived biochars have been evaluated for removal of toxic metals in numerous studies. The results of these studies highlighted the capability of biochars as great potential low cost sorbents and indicated the important role of biochar's physiochemical characteristics in its uptake ability [2, 7, 13–16].

Annually, enormous amounts of biowastes from oil palm mills are produced around the world which contribute to great environmental concern, and many researchers have been focused on conversion of these wastes into value-added products. Oil palm empty fruit bunches account for 23% per ton of fresh fruit bunch which should be handled properly [17]. Currently, the majority of empty fruit bunches are combusted in the incinerators for the purpose of fertilizer production which produces “white smoke” and considered an environmental concern by the Department of Environment [18]. The great amount of generated empty fruit bunches from oil palm mills could be a potential feedstock substrate for biochar production by an environmentally friendly method. Production of biochar from oil palm empty fruit bunches, an abundant waste of oil palm mill, has twofold advantage: firstly, production of low cost and ecofriendly adsorbent for removal of heavy metals and, secondly, solving part of waste disposal problem by conversion of unwanted wastes into value-added products.

Production of efficient sorbent for this purpose has been always a concern. Among the sorbent characteristics, surface area, surface functionalities, and acceptable level of yield are important in adsorption process design [19]. These characteristics of biochar are controlled by its production conditions and primary feedstock properties [19]. In assessing the effect of production conditions, employing an adequate experimental design is key point.

Design of experiment (DOE) enables engineering researchers to alleviate the costs by rationalizing the experiments and improving productivity and product quality. Response surface methodology, one of the DOE techniques, is applied for experimental design, statistical modeling, and optimization of a process. It is a helpful tool in studying the effect of factors and their interactions on specific response to optimize the response of interest [20].

RSM has been widely used in optimization of experimental conditions of various processes; however, its application in production of biochar is very rare in literature. Some previous studies focused on applying RSM in determining the effect of different parameters during production of adsorbent and batch adsorption experiments, on removal of hazardous contaminants such as chromium [21, 22], copper [23], nickel [24], reactive blue dye [25], lead and zinc [26], and cationic and anionic dye [27].

To our knowledge, no study has been performed on optimized production of biochar from oil palm empty fruit bunches, applying RSM. Therefore, the focus of the present study is to produce biochar from oil palm empty fruit bunches and to optimize the preparation conditions using central composite design for yield and its adsorption capacity towards zinc. The influence of three numerical variables, namely, highest treatment temperature, heating rate, and residence time, on the responses was considered, simultaneously.

## 2. Materials and Methods

### 2.1. Preparation of Biochar

Oil palm empty fruit bunches were collected from Ulu Langat Palm Oil Mill, a local oil palm mill in Malaysia; Seri Sdn Bhd (Lot 3115, Batu 34, Jalan Banting, Dengkil, 43800, Selangor, Malaysia). The biomass samples were dried in oven at 105°C for 24 h to get constant weight. Afterward, the samples were cut into smaller parts (2 nm) to ease the pyrolysis process. The pyrolysis process was performed by placing samples in vertical stainless steel reactor and heating from room temperature to predetermined temperature with specific heating rate and they were kept at highest temperature for specific duration. Purified nitrogen (99.995%) with flow rate of 150 cm^3^/min was used during pyrolysis process to wash tarry vapors away from the surface of biochar.

### 2.2. Design of Experiment

Response surface methodology (RSM) is a statistical technique which utilizes the quantitative data of the experiments for purpose of determining the regression model and optimum operation conditions [28]. It applies mathematical and statistical methods for analyzing and simulating of a process with the objective of optimizing a response which is affected by several independent variables [29]. Central composite design (CCD) is the most common method for fitting quadratic surfaces and optimization with minimum number of experiments [30]. In general, CCD consists of 2^*n*^ factorial runs, 2*n* axial runs, and *n*_*c*_ center points in which *n* is the number factors.

Three numerical factors have been considered for this purpose, namely, *x*_1_ as highest treatment temperature (HTT), *x*_2_ as heating rate (HR), and *x*_3_ as residence time (RT). Two responses in this work were yield (*Y*_1_) and adsorption capacity of OPEFB biochar (*Y*_2_). Number of runs based on CCD method for three independent variables is equal to 20 experiments, including 8 factorial points, 6 axial points, and 6 center points based on the following equation [31]:

(1) $$\begin{array}{c} N=2^{n}+2n+n_{c}=2^{3}+2\times 3+6=20, \end{array}$$

where *N* is the total number of experiments and *n* is the number of factors. The center points are useful in considering the errors of experiments and reproducibility of the model. For all three factors the ranges have been entered based on “−alpha” and “+alpha” level where alpha is the distance of axial points from center points, intending not to have any unreachable level for the factors. These variables and their respective ranges were selected based on screening tests in preliminary studies. Levels of independent variables are given in Table 1. The experiments were conducted as randomized in order to minimize the influence of uncontrolled factors. An empirical model was developed to correlate the three independent variables and each response based on second-order polynomial equation as follows:

(2) $$\begin{array}{c} Y=b_{0}+\overset{n}{\underset{i=1}{\sum }}b_{i}x_{i}+{\left(\;\sum_{i=1}^{n}b_{ii}x_{i}\;\right)}^{2}+\overset{n-1}{\underset{i=1}{\sum }}\overset{n}{\underset{j=i+1}{\sum }}b_{ij}x_{i}x_{j}. \end{array}$$

In which, *b*_0_ is the constant, *b*_*i*_ is the linear coefficient and *b*_*ii*_ is the quadratic coefficient, and *x*_*i*_ and *x*_*j*_ are the coded values of pyrolysis conditions.

### 2.3. Model Fitting and Statistical Analysis

The analysis of experimental data was performed using Design Expert version 7 (STAT-EASE Inc., Minneapolis, USA) for regression analysis to fit the empirical models and statistical significance evaluation of the models.

### 2.4. Adsorption Experiments

Zinc stock solution was prepared by dissolving appropriate amount of ZnCl_2_ (Anhydrous, Sigma Aldrich) in Millipore water with purity of 99.99% to concentration of 2000 ppm. The batch adsorption experiments were conducted in 20 sets of 250 ml Erlenmeyer flasks. In a typical experiment, 0.4 g of biochar was added to 50 ml of heavy metal solution with concentration of 200 ppm and the pH of solution was set at 6. Subsequently the mixture was agitated for 24 hours for equilibration and then filtered employing Whatman filter paper. The equilibrium time was selected based on preliminary studies as the time when the zinc concentration remained constant. The resultant solution was analyzed for concentration of zinc utilizing A Analyst 400 PerkinElmer atomic absorption spectrometer device. The adsorption capacity of biochars was calculated by the following equation:

(3) $$\begin{array}{c} Q=\frac{\left(C_{i}-C_{e}\right)v}{m}. \end{array}$$

In the previous equation, *C*_*i*_ and *C*_*e*_ are the initial and equilibrium concentration of zinc (mg/l), respectively. *Q* (mg/g) is the adsorption capacity of biochar, *m* is the dry mass of biochar (g), and *v* is for volume of solution (l).

### 2.5. Yield of Biochar

The yield of biochar was calculated according to the following equation:

(4) $$\begin{array}{c} Yield=\frac{\text{weight of biochar}\left(\text{g}\right)}{\text{weight of dry biomass}\left(\text{g}\right)}*100. \end{array}$$

### 2.6. Characterization of Optimum Biochar

The surface structure of biochar was analyzed by means of scanning electron microscopy utilizing Zeiss scanning electron microscope (Carl Zeiss Germany). *N*_2_ adsorption at 77 K was performed for surface area and pore volume estimation of biochar, using Sorptomatic 1990 system (Thermo Finnigan). Surface functional groups of biochars were determined with aid of Fourier Transform Infrared spectroscopy, using Nicolet Nexus 6700 FTIR spectrometer. The KBr pellet was prepared by mixing well grinded biochar samples with KBr powder at ratio of 1 : 100 approximately.

## 3. Results and Discussion

### 3.1. Development of Regression Model Equation

The complete design matrix of experiments with the obtained results for both responses is presented in Table 2. A polynomial regression equation was developed based on CCD to analyze the variables, their interactions, and identifying the significant factors. Runs 1, 4, 12, 15, 16, and 19 are center points and used to determine the error of experiments. Biochar yield was found to be in the range of 23.2% and 33.73% while the adsorption capacity for zinc obtained ranged between 7.59 mg/g and 14.74 mg/g.

Based on the sequential model sums of squares, the fitted model was chosen as the highest order polynomial model in which the additional terms were significant and model was not aliased. For both responses, the quadratic model was selected as suggested by the software. The final empirical equations for yield (*Y*_1_) and zinc adsorption capacity of biochar (*Y*_2_) in terms of coded variables are presented in (1) and (2), respectively.

(5) Y2=25.91−2.39x1−1.23x2−0.61x3+0.40x1x2+0.23x1x3−0.22x2x3+1.15x12+0.24x22−0.051x32,

(6) Y2=14.01+1.56x1−0.66x2+0.90x3+0.11x1x2+0.28x1x3+0.035x2x3−1.12x12−0.56x22−1.07x32.

Positive sign in front of the terms represents the synergistic influence while negative sign represents antagonistic influence. Values of coefficient determination, *R* squared, adjusted *R* squared, standard deviation (SD), and coefficient of variation (CV) were used to evaluate the quality of developed model. *R*^2^ values for (5) and (6) were 0.9766 and 0.9794, implying that models were able to explain 97.66% and 97.94% of total variance in biochar yield and biochar adsorption capacity for zinc, respectively. The closer *R*^2^ is to unity, the better the model fits experimental data. Both *R*^2^ values are considered relatively high and suggesting satisfactory agreement between model and experimental data. The adjusted *R* squared with values of 0.9556 and 0.9609 for (5) and (6), respectively, indicates good sample size and ability of model. Coefficient of variation (CV) is a measure of the model reproducibility and considered as the ratio of standard deviation to mean value of observed response. The model is regarded as reproducible if the value of CV for the model is less than 10% [26, 32]. Coefficients of variation (CV) for both studied responses were less than 10% and were equal to 2.04% and 3.47% for *Y*_1_ and *Y*_2_, respectively. The standard deviation values for the models were 0.55 and 0.42 which reflect the accuracy of the model. The adequacy of the model was further checked by analysis of variance (ANOVA). The ANOVA for quadratic model for yield of biochar is given in Table 3. From ANOVA for yield of biochar, the *F*-value was 46.44 and *p* value was less than 0.0001, reflecting that model was significant. Regarding the model terms, *p* value less than 0.05 implies that model term was significant. According to Table 3 for yield of biochar, *x*_1_, *x*_2_, *x*_3_, and *x*_1_^2^ were significant model terms, whereas *x*_1_*x*_2_, *x*_2_*x*_3_, *x*_1_*x*_3_, *x*_2_^2^, and *x*_3_^2^ were insignificant terms to the model.

The result of ANOVA for quadratic model for adsorption capacity of biochar is presented in Table 4. The *F*-value of 52.95 with *p* value less than 0.0001 indicates the significance of the model. Table 4 illustrates that *x*_1_, *x*_2_, *x*_3_, *x*_1_^2^, *x*_2_^2^, and *x*_3_^2^ are significant model terms. On the other hand, the interactions of factors *x*_1_*x*_2_, *x*_1_*x*_3_, and *x*_2_*x*_3_ were insignificant terms to the response.

**Figure 1:**
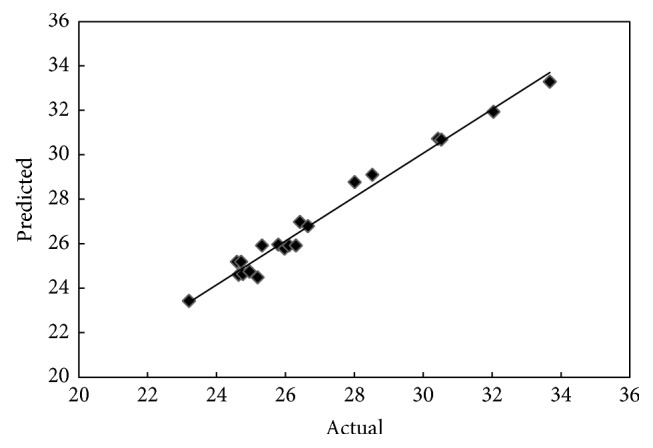
Predicted versus actual values for yield of OPEFB biochar.

**Figure 2:**
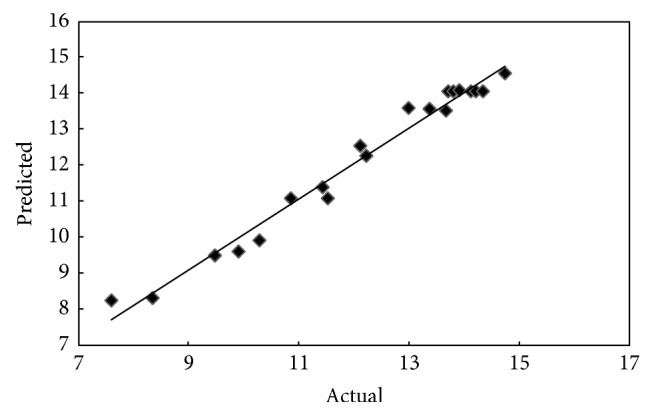
Predicted versus actual values for adsorption capacity of OPEFB biochar.

The predicted versus experimental values for yield and adsorption capacity of OPEFB biochar are illustrated in Figures 1 and 2, respectively. As it can be observed, the predicted values are close to the experimental values which indicate that the developed model successfully fitted the correlation between variables and responses.

### 3.2. Yield of Oil Palm Empty Fruit Bunches Biochar (OPEFBB)

**Figure 3:**
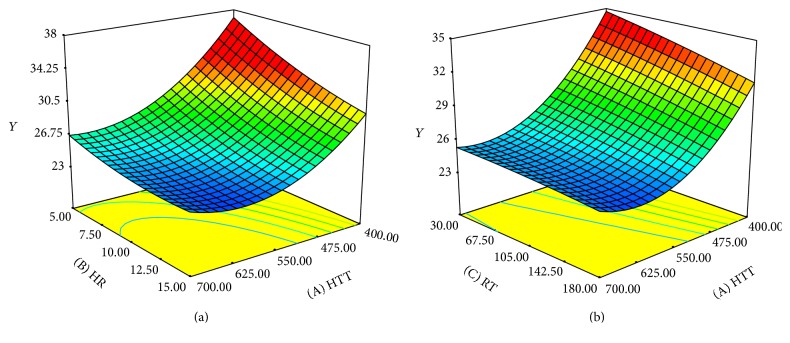
(a) Surface plot of percentage of yield (*Y*) as a function of highest treatment temperature (HTT) and heating rate (HR) at fixed residence time of 105 minutes. (b) Surface plot of percentage of yield (*Y*) as a function of highest treatment temperature (HTT) and residence time (RT) at fixed heating rate of 10°C per minute.

Referring to the yield of biochar, HTT had the greatest influence on the response followed by the HR and RT. Figures 3(a) and 3(b) represent the three-dimensional response surfaces to demonstrate the influence of biochar preparation conditions on yield.  Figure 3(a) illustrates the surface plot of percentage of yield under the impact of highest treatment temperature (HTT) and heating rate (HR) where residence time (RT) was fixed at zero level (105 minutes). On the other hand, Figure 3(b) illustrates the effect of highest treatment temperature and residence time on yield (heating rate was fixed at zero level). As demonstrated in Figures 3(a) and 3(b), the biochar yield decreased with increasing in HTT, HT, and RT.

Similar trend was also reported in other works, studying the influence of production parameters on char yield. Al-Wabel et al. reported reduction in yield of* Conocarpus* wastes biochar by increasing the highest treatment temperature, specifically when the temperature increased more than 200°C. This may be due to the destruction of cellulose and hemicellulose and combustion of organic matters [33]. Angin found that the yield of safflower seed cake-based biochar was reduced by rising pyrolysis temperature and heating rate. The effect of heating rate on the yield was also reported to be more significant at lower pyrolysis temperatures [34]. McBeath et al. examined the effect of pyrolysis conditions on yield and characteristics of biochar from eighteen different feedstocks and concluded that increasing pyrolysis temperature resulted in lower yield of char [35]. This is due to the evaporation of volatile matters and higher heat and mass transfer rate and destructive reactions. In another study by Hmid et al. both pyrolysis temperature and heating rate were reported as influential factors on the yield of biochars derived from olive solid residues considerably [36]. Ronsse et al. studied the influence of pyrolysis peak temperature and residence time on the yield of biochars from various feedstocks. It was observed that biochar yield tends to decrease by increasing residence time and peak temperature [37].

In this work, all three production variables correlated negatively with biochar yield. The interaction effect of production parameters on biochar yield was not significant. The increase in pyrolysis temperature resulted in releasing of more volatile compound, primary decomposition of parent material, and possible secondary decomposition of produced biochar. Increasing in heating rate may cause severe heat and mass transfer rate which led to lower biochar yield. The influence of residence time on biochar yield was not also significant.

### 3.3. Adsorption Capacity of Oil Palm Empty Fruit Bunches Biochar (OPEFBB)

**Figure 4:**
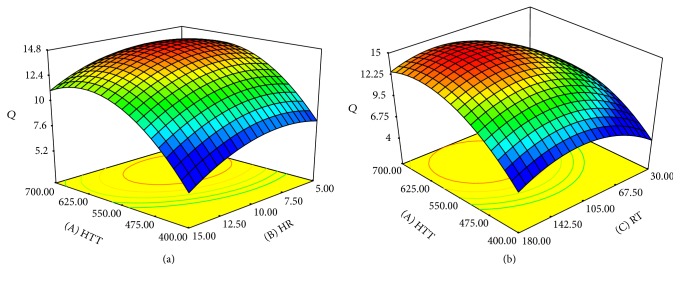
(a) Surface plot of adsorption capacity of biochar (*Q*) as a function of highest treatment temperature (HTT) and heating rate (HR) at fixed residence time of 105 minutes. (b) Surface plot of adsorption capacity of biochar (*Q*) as a Function of highest treatment temperature (HTT) and residence time (RT) at fixed heating rate of 10°C per minute.

Based on the ANOVA, all three variables and their quadratic effect were found to be significant on the adsorption capacity of OPEFB biochar; however, HTT with *F*-value of 33.4246 was the most influential factor. Figures 4(a) and 4(b) demonstrate the three-dimensional response surfaces to show the influence of operating variables on adsorption capacity. The effect of the highest treatment temperature and heating rate on adsorption capacity where residence time was maintained at zero level is depicted in Figure 4(a), whereas the effect of highest treatment temperature and residence time on adsorption capacity of OPEFB biochar when heating rate was fixed at zero level is presented in Figure 4(b). As it can be seen from Figure 4(a), the adsorption capacity of biochar increases with increasing temperature and heating rate up to specific point and, afterward, it decreases possibly because of blockage of some pores due to melting and releasing of tars. The highest treatment temperature had a positive linear effect on adsorption capacity, whereas heating rate has a negative effect. In addition, both factors have a negative quadratic effect on adsorption capacity of OPEFB biochar. Raising heating rate increases the adsorption capacity up to a certain point after which it reduces. This is due to the fact that the time for releasing volatiles becomes shortened at high heating rate which results in agglomeration of volatiles between and inside the pores and, therefore, the chance of blocking the pores entrance increases. Similar results have been reported in other studies [34, 38, 39].

Surface plot of the effect of highest treatment temperature and residence time on adsorption capacity of OPEFB biochar when heating rate was fixed at zero level is presented in Figure 4(b). Residence time increase favors the adsorption capacity up to a certain point of time, after which further increase causes the adsorption capacity to decrease due to the failure of pore walls' strength and their destruction.

### 3.4. Process Optimization

Getting high yield is an important factor in producing biosorbents, but adsorption capacity determines the quality of product. Therefore, both high yield and high adsorption capacity is desirable for economics feasibility of the product. However, optimizing both of these responses is very difficult as their affecting factors are opposite, which means adsorption capacity of OPEFB biochar increases while the yield decreases and vice versa. Consequently, in order to compromise between the two responses, the function of desirability has been employed using Expert-Design software version 7 (STAT-EASE Inc., Minneapolis, USA). The experimental conditions which showed the highest desirability were chosen for verification. The yield and adsorption capacity of the biochar prepared under optimum conditions compared to the predicted values are presented in Table 5. The optimum biochar from oil palm empty fruit bunches was obtained using highest temperature of 615°C, heating rate of 8°C/min, and residence time of 128 min. The optimum biochar showed the adsorption capacity of 15.03 mg/g towards zinc and the biochar yield of 25.27%.

As it can be observed from Table 5, the obtained experimental results are in good agreement with model prediction points with relatively small deviation, indicating the accuracy of the model. Overall, OPEFB proved to be a potential promising substrate for production of biochar, a green low cost sorbent, with high performance for removal of heavy metals (zinc) from the aqueous solution.

### 3.5. Characterization

**Figure 5:**
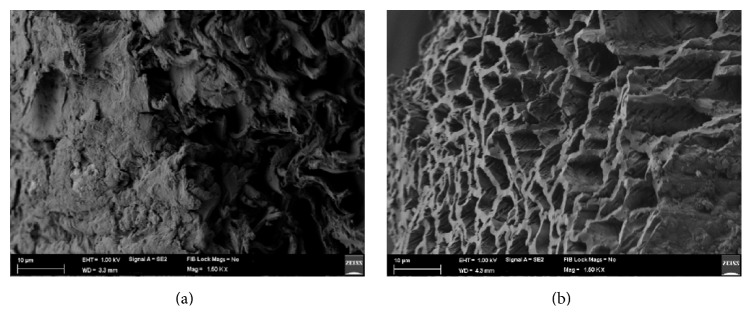
(a) SEM image of oil palm empty fruit bunches. (b) SEM image of OPEFB biochar produced under optimum conditions.

Figures 5(a) and 5(b) illustrate the scanning electron microscope images of the precursor (OPEFB) and the biochar obtained under optimum conditions. As it is clear in the micrograph, the external surfaces of the biochar obtained under optimized conditions consist of cracks, crevices, and significant amount of honey comb like pores with various sizes. Comparison of this micrograph with SEM micrograph of OPEFB revealed that during pyrolysis the cracks and pores of biochar become cleaner due to increase in devolatilization and, therefore, more ordered structural arrangement can be detected in optimum product.

**Figure 6:**
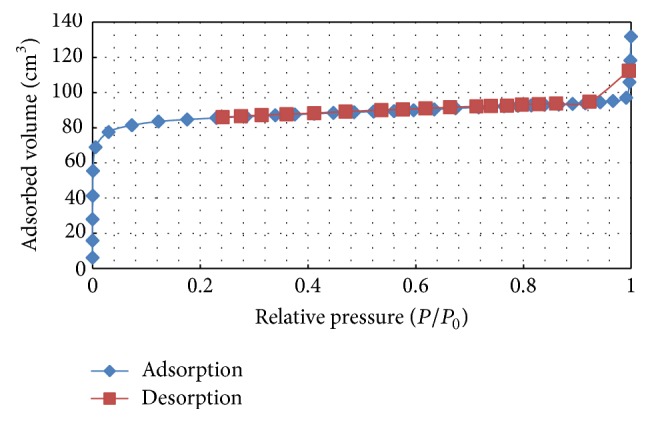
Adsorption-desorption isotherm graph of OPEFB biochar produced at RSM optimum conditions.

Figure 6 displays the adsorption-desorption isotherm of OPEFB biochar synthesized under RSM optimum conditions. This adsorption isotherm can be classified as type I with a type H4 hysteresis loop at relative pressure 0.9, resembling microporous structured materials with some degree of mesoporosity. The surface physical parameters determined from N2 adsorption isotherm for optimum product and OPEFB biochar synthesized at 300°C from the preliminary studies are summarized in Table 6. By comparison of the results of textural properties of optimum OPEFB biochar with unoptimized one, it is evident that RSM optimum product displays higher BET surface area, micropore surface area, micropore volume, and mesopore volume indicating pore development at optimum pyrolysis conditions. This is most likely due to the progressive decomposition of volatile matter and better carbonization that leads to enhanced porosity.

## 4. Conclusion

In this study, novel, low cost adsorbent from empty fruit bunches were synthesized by pyrolysis. The influence of pyrolysis conditions on the yield and adsorption capacity of oil palm empty fruit bunches biochar for zinc was studied employing RSM. Through analysis of developed response surfaces, HTT was found to have the most significant influence on both responses. The optimum biochar was obtained at highest treatment temperature of 615°C, heating rate of 8°C/min, and residence time of 128 min which demonstrated 15.18 mg/g zinc adsorption capacity and 25.49% yield. The resultant biochar produced under optimum conditions demonstrates 421.26 m^2^/g surface area and 0.15 cm^3^/g total pore volume.

## Figures and Tables

**Table 1:** Level of different factors.

Variables	Units	(−1) level	(+1) level	−alpha	+alpha
(A) HTT	(°C)	460.8095	639.1905	400	700
(B) HR	(°C/min)	7.026982	12.97302	5	15
(C) RT	(min)	60.40473	149.5953	30	180

**Table 2:** Design matrix with results.

Run	Point type	(A) HTT (°C)	(B) HR (°C/min)	(C) RT (min)	Yield (%)	*Q* (mg/g)
1	Center	550.00	10.00	105.00	26.14	13.72
2	Axial	550.00	15.00	105.00	25.2	11.43
3	Fact	460.81	7.03	60.40	32.08	10.28
4	Center	550.00	10.00	105.00	25.97	13.91
5	Axial	550.00	10.00	180.00	24.93	12.11
6	Axial	550.00	10.00	30.00	26.67	9.47
7	Axial	700.00	10.00	105.00	24.67	13.67
8	Fact	639.19	7.03	149.60	26.01	14.74
9	Axial	400.00	10.00	105.00	33.73	7.59
10	Fact	460.81	12.97	149.60	26.45	9.89
11	Axial	550.00	5.00	105.00	28.06	13.01
12	Center	550.00	10.00	105.00	26.25	14.22
13	Fact	460.81	12.97	60.40	28.54	8.35
14	Fact	460.81	7.03	149.60	30.52	11.53
15	Center	550.00	10.00	105.00	25.88	14.13
16	Center	550.00	10.00	105.00	25.9	13.81
17	Fact	639.19	7.03	60.40	26.29	12.22
18	Fact	639.19	12.97	149.60	23.2	13.37
19	Center	550.00	10.00	105.00	25.3	14.36
20	Fact	639.19	12.97	60.40	24.71	10.86

**Table 3:** Analysis of variance (ANOVA) for response surface quadratic model for yield.

Source of data	Sum of squares	Degree of freedom (DF)	Mean square	*F*-value	*p* value Prob > *F*	Comment
Model	125.62	9	13.96	46.44	<0.0001	Significant
*x*_1_	77.90	1	77.90	259.18	<0.0001	
*x*_2_	20.69	1	20.69	68.84	<0.0001	
*x*_3_	5.13	1	5.13	17.05	0.0020	
*x*_1_*x*_2_	1.30	1	1.30	4.31	0.0646	
*x*_1_*x*_3_	0.43	1	0.43	1.44	0.2580	
*x*_2_*x*_3_	0.39	1	0.39	1.29	0.2828	
*x*_1_^2^	19.09	1	19.09	63.50	<0.0001	
*x*_2_^2^	0.85	1	0.85	2.81	0.1244	
*x*_3_^2^	0.038	1	0.038	0.13	0.7302	
Residual	3.01	10	0.30			
Lack of fit	2.46	5	0.49	4.51	0.0618	Not significant
Pure error	0.55	5	0.11			

**Table 4:** Analysis of variance (ANOVA) for response surface quadratic model for adsorption capacity of OPEFB biochar.

Source of data	Sum of squares	Degree of freedom (DF)	Mean square	*F*-value	*p* value Prob > *F*	Comment
Model	84.47	9	9.39	52.95	<0.0001	Significant
*x*_1_	33.42	1	33.42	188.57	<0.0001	
*x*_2_	5.87	1	5.87	33.14	0.0002	
*x*_3_	11.01	1	11.01	62.09	<0.0001	
*x*_1_*x*_2_	0.09	1	0.09	0.5	0.4967	
*x*_1_*x*_3_	0.63	1	0.63	3.54	0.0894	
*x*_2_*x*_3_	0.01	1	0.01	0.06	0.8189	
*x*_1_^2^	18.2	1	18.2	102.69	<0.0001	
*x*_2_^2^	4.55	1	4.55	25.65	0.0005	
*x*_3_^2^	16.42	1	16.42	92.62	<0.0001	
Residual	1.77	10	0.18			
Lack of fit	1.46	5	0.29	4.65	0.0585	Not significant
Pure error	0.31	5	0.06			

**Table 5:** Model validation.

Model desirability	HTT (*x*_1_)	HR (*x*_2_)	RT (*x*_3_)	Biochar yield (%)		Adsorption capacity (mg/g)	
				Predicted	Experimental	Predicted	Experimental
0.934	615	8	128	25.28	25.49	14.98	15.18

**Table 6:** Results of surface area and pore characterization of optimized RSM OPEFB biochar.

Material	BET surface area (m^2^/g)	Micropore surface area (m^2^/g)	Micropore volume (cm^3^/g)	Mesopores volume (cm^3^/g)	Total pore volume (cm^3^/g)	Average pore diameter (Å)	Reference
RSM optimized OPEFB biochar	421.26	347.09	0.13	0.018	0.15	14.41	Present study
OPEFB biochar (300°)	44.38	7.80	0.003	0.317	0.32	28.84	Preliminary study
